# An Endoscopic Nasomediastinal Approach to a Mediastinal Abscess Developing after Zenker's Diverticulectomy

**DOI:** 10.1155/2017/8726706

**Published:** 2017-08-02

**Authors:** Fatih Altintoprak, Kemal Gundogdu, Ahmet Tarik Eminler, Erkan Parlak, Guner Cakmak, Yener Uzunoglu Mustafa

**Affiliations:** ^1^Department of General Surgery, Faculty of Medicine, Sakarya University, Sakarya, Turkey; ^2^Department of General Surgery, Sakarya University Research and Educational Hospital, Sakarya, Turkey; ^3^Department of Gastroenterology, Faculty of Medicine, Sakarya University, Sakarya, Turkey

## Abstract

Zenker's diverticulum is the most frequent symptomatic diverticulum of the esophagus, but the prevalence is <0.1%. The optimal treatment is surgery. Here, we present a nasomediastinal drainage approach to treatment of a mediastinal abscess, developing in the late postoperative period and attributable to leakage from the staple line.

## 1. Introduction

Zenker's diverticulum, a pseudodiverticulum of the esophagus, requires surgical treatment; the classical approach is diverticulectomy and cricopharyngeal myotomy [1]. Potential complications include leakage, fistulation, an abscess, mucosal perforation, mediastinitis, hemorrhage, cervical hematoma, and recurrent nerve injury [2]. Secondary interventions may be needed to treat complications developing in the early postoperative period. Here, we report a favorable outcome after fistulation and abscess formation was observed in the late postoperative period, attributable to sutural leakage. We drained the abscess using an endoscopic nasomediastinal approach; we also prescribed hydration therapy and antibiotics.

## 2. Case Presentation

**Figure 1:**
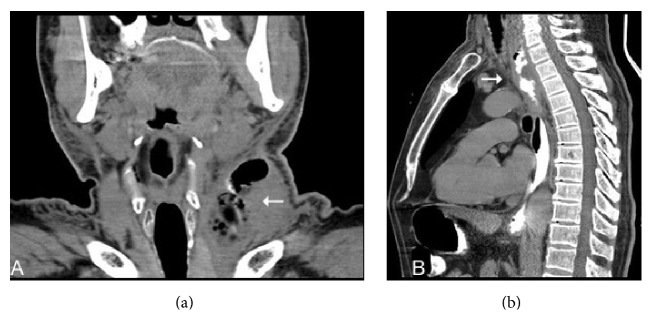
Computed tomography (CT) findings. (a) Inflammation and an abscess on the left side of the neck (arrow). (b) Inflammation of and contrast leakage into the left anterior mediastinum (arrow).

**Figure 2:**
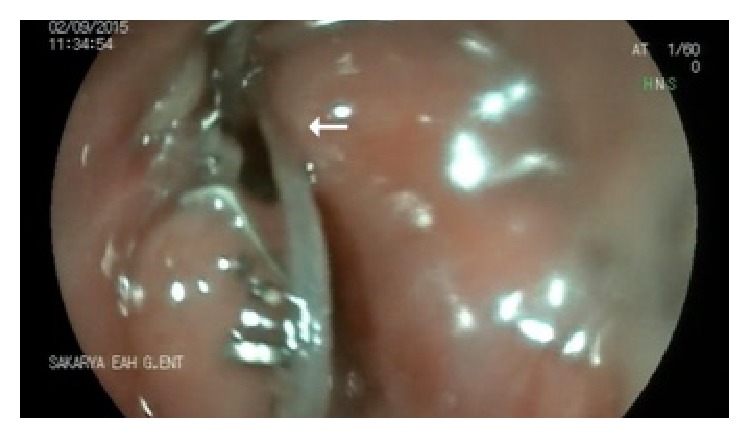
Endoscopic finding. A gap 0.5 × 1 cm in dimensions was evident in the region of the diverticulectomy (arrow).

**Figure 3:**
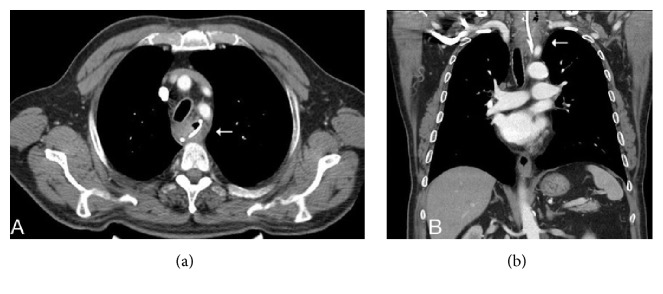
CT images obtained after placement of an endoscopic catheter, which was confirmed to be correctly located ((a) and (b), arrows).

A 66-year-old male presented with diverticulectomy and cricopharyngeal myotomy. He was diagnosed with Zenker's diverticulum and discharged 7 days after an uneventful operation. He re-presented 27 days later with fever, dysphagia, and inflammation of the cervical incision line. On cervical ultrasonography, an abscess 5 × 4 cm in dimensions was evident under the incision; the anastomosis was leaking into the esophagus. Cervical and thoracic computed tomography (CT) revealed inflammation in the left side of the neck and an abscess 53 × 36 mm in dimensions in the left anterior mediastinum (Figure 1). We prescribed antibiotics and parenteral hydration; the cervical abscess drained spontaneously. Endoscopically, a gap 0.5 × 1 cm in dimensions was detected in the region of the diverticulectomy (Figure 2). We were unable to place an endoscopic clip. We placed a no. 7 French nasobiliary drainage catheter through the gap and then into the mediastinum using a guide and confirmed via CT that the placement was correct (Figure 3). Initially, about 50 mL of fluid was drained daily; this gradually decreased and ceased on day 11. On day 13, a repeat radiological examination showed that the leakage, cervical inflammation, and mediastinal abscess had regressed. The nasomediastinal drain was removed on day 15. After oral intake was resumed, the patient was discharged on day 17 and no problem has emerged to the time of the 4-month follow-up.

## 3. Discussion

Zenker's diverticulum is a hypopharyngeal pouch caused by dehiscence-mediated herniation of mucosal and submucosal layers of the esophagus at the triangle of Killian (a weak area lying between the transverse fibers of the cricopharyngeus muscle and the oblique fibers of the lower inferior constrictor muscle) [3]. As a pharyngoesophageal/cricopharyngeal diverticulum, Zenker's diverticulum is the most frequent symptomatic diverticulum of the upper gastrointestinal tract. Zenker's diverticulum is a pulsion diverticulum developing secondary to a lack of compliance of the cricopharyngeal muscle and the upper esophageal sphincter zone. The therapeutic principle is based on the underlying pathophysiology and features extramucosal myotomy of the cricopharyngeal muscle and the upper esophageal sphincter zone; this is the most crucial step in treatment [4, 5]. In terms of surgical options, no surgery may be required when the diverticulum is small (<2 cm in diameter), but larger diverticula require either resection, diverticulectomy, or diverticulopexy with cricopharyngeal myotomy [6]. Although various transoral, endoluminal treatment modalities have been described in recent years, the classical surgical treatment approach remains the most commonly used [7]. We performed classical surgery via a cervical incision.

Possible postoperative problems include paralysis of the laryngeus recurrens nerve, esophageal stenosis, an abscess, mediastinitis, a pharyngocutaneous fistula, hematoma, and esophageal perforation; all require further intervention [6]. A major complication is leakage from, or fistulation of, the surgical site. Such leaks have been reported in 1–20% of patients [7]. Anastomotic leakage generally develops in the early postoperative period and prolongs the hospital stay. Here, we were confronted with late leakage developing after discharge.

Various minimally invasive endoscopic treatments for esophageal leakage have been described in the literature. These include endoscopic clipping, stenting, and drainage, as well as vacuum-assisted closure. We describe a nonsurgical intervention used to treat a late complication (abscess formation attributable to anastomotic leakage evident on esophageal imaging 27 days after diverticulectomy and cricopharyngeal myotomy). We chose to perform endoscopic nasomediastinal drainage, which was successful. Thus, there was no need for a possibly debilitating secondary surgical intervention.

## 4. Conclusion

Late leakage and abscess formation are very rare complications after Zenker diverticulectomy. Postoperative fever, a cervical mass, and an abscess are suggestive of leakage; in such cases the patient requires close follow-up. Drainage via an endoscopic nasomediastinal catheter is a feasible alternative to surgery in suitable patients.
